# *WavePropaGator*: interactive framework for X-ray free-electron laser optics design and simulations^1^

**DOI:** 10.1107/S160057671600995X

**Published:** 2016-07-06

**Authors:** Liubov Samoylova, Alexey Buzmakov, Oleg Chubar, Harald Sinn

**Affiliations:** a European XFEL GmbH, Albert-Einstein-Ring 19, Hamburg, 22761, Germany; b Institute of Crystallography, Leninskii prospekt 59, Moscow, 119333, Russian Federation; c National Synchrotron Light Source II, Brookhaven National Laboratory, Upton, NY 11973, USA

**Keywords:** wavefront propagation, X-ray free-electron lasers, XFELs, Fourier optics, start-to-end simulations, data analysis and visualization, computer programs

## Abstract

This article describes the *WavePropaGator* (*WPG*) package, a new interactive software framework for coherent and partially coherent X-ray wavefront propagation simulations. The package has been developed at European XFEL for users at the existing and emerging free-electron laser (FEL) facilities, as well as at the third-generation synchrotron sources and future diffraction-limited storage rings. The *WPG* addresses the needs of beamline scientists and user groups to facilitate the design, optimization and improvement of X-ray optics to meet their experimental requirements. The package uses the *Synchrotron Radiation Workshop* (*SRW*) C/C++ library and its Python binding for numerical wavefront propagation simulations. The framework runs reliably under Linux, Microsoft Windows 7 and Apple Mac OS X and is distributed under an open-source license. The available tools allow for varying source parameters and optics layouts and visualizing the results interactively. The wavefront history structure can be used for tracking changes in every particular wavefront during propagation. The batch propagation mode enables processing of multiple wavefronts in workflow mode. The paper presents a general description of the package and gives some recent application examples, including modeling of full X-ray FEL beamlines and start-to-end simulation of experiments.

## Abstract 2

The *WavePropaGator* (*WPG*) package is a new interactive cross-platform open-source software framework for modeling of coherent and partially coherent X-ray wavefront propagation. The *WPG* addresses the needs of beamline scientists and user groups to facilitate the design, optimization and improvement of X-ray optics to meet their experimental requirements. The paper presents a general description of the package and gives some recent application examples.

## 1. Glossary   

FEL: free-electron laser.

FFT: fast Fourier transform.

Git: a distributed revision control and source code management system (http://git-scm.com).

GitHub: a web-based Git repository hosting service (https://github.com/).

HDF5: Hierarchical Data Format. To view HDF5 files one can use the official free *HDFView* tool (https://www.hdfgroup.org/products/java/hdfview).

*IPython Notebook*: an interactive computational environment for combining code execution, rich text, mathematics, plots and rich media (http://ipython.org/notebook.html).

MPI: Message Passing Interface. A standardized and portable message-passing system designed by a group of researchers from academia and industry to function on a wide variety of parallel computers (http://www.mpi-forum.org/).

SASE: self-amplified spontaneous emission. The process whereby an electron beam passes through a long undulator, such that the initial random field of spontaneous radiation becomes amplified in intensity and enhanced in coherence characteristics. SASE FEL pulses are characterized by spiky structure and fluctuations in wavelength and FEL pulse energy.

## 2. Introduction   

The X-ray free-electron laser facilities (XFELs) that have emerged in recent years deliver radiation with a unique combination of properties, such as extreme peak field intensities, ultra-short X-ray pulse duration and a high degree of transverse coherence of the XFEL radiation, and thus establish a new specific combination of requirements for X-ray optics. Additional factors such as the varying beam size and divergence as a function of the electron bunch charge, the very large distances to the source, and, as a consequence, the large apertures required for the optics further complicate the environment.

Besides, the end stations at XFEL facilities have very versatile layouts, which can include multi-crystal monochromators, split-and-delay lines and units for pump–probe experiments (Roling *et al.*, 2014 ▸), monochromators for hard and soft X-ray self-seeding schemes (Amann *et al.*, 2012 ▸; Inagaki *et al.*, 2014 ▸), and various refocusing optics. For a correct optics design, one has to analyze and optimize all the factors that have an impact on the beam properties after propagation of the beam through the optics. Hard X-ray FEL wavefronts are subject to distortions caused by imperfections of the optics, in particular the effect of mirror surface errors, which have been measured experimentally at LCLS and SACLA (Rutishauser *et al.*, 2012 ▸; Kayser *et al.*, 2014 ▸). Moreover, characterizing focused XFEL intense wavefields is crucial for their use in single-shot diffractive imaging experiments (Loh *et al.*, 2013 ▸; Park *et al.*, 2013 ▸).

The wave optics formalism is a natural and reliable way to deal with interference effects that are unavoidable in coherent beam propagation (Bahrdt *et al.*, 2014 ▸; Chubar *et al.*, 2011 ▸). We present here the *WavePropaGator* (*WPG*) package, the goal of which is to provide a user-friendly software environment that any scientist involved in XFEL optics design can use, without having specific experience in numerical wave optics calculations. The package is built on top of the open-source *Synchrotron Radiation Workshop* (*SRW*) software (Chubar *et al.*, 2011 ▸), which is actively used for development of X-ray and infrared beamlines at synchrotron light sources. The *SRW* code is based on the principle of physical optics using FFT and asymptotic expansion based propagators and is capable of accurate modeling of fully and partially coherent synchrotron radiation emission and propagation through different types of optics. The software is able to calculate the propagation of SASE XFEL pulses, which requires much larger computing resources than propagation of steady state beams or longitudinally coherent pulses.

For the *WPG* framework we use Python structures, which provide the possibility for coding in self-explaining meta-language, so that a complete beamline with tens of optical elements can be specified within one page of text. One can easily create and modify such scripts without going into implementation details. In addition, an experienced user can create propagators for new optics types in Python and include them in the beamlines. The *WPG* framework scripts can be run either within the *IPython Notebook* interactive environment, combining code execution, rich text, mathematics and plots in one file, or in batch file mode, as a Python script in Python or an IPython shell. The *WPG* also includes modules necessary for start-to-end simulations of XFEL experiments, which simulate propagation of the XFEL pulses (Manetti *et al.*, 2016 ▸) through beamline optics and provide an interface to codes modeling the interaction with the sample (*SingFEL*, *PMI*; Yoon *et al.*, 2016 ▸).

## 3. Coherent wavefront propagation   

This section summarizes the basics of the calculation methods implemented in the *SRW* (Chubar & Elleaume, 1998 ▸; Chubar *et al.*, 2002 ▸, 2008 ▸, 2011 ▸) that are most relevant for FEL applications and are available through the *WPG* interface.

For small emission and observation angles, the propagation of the transverse components in free space from a point **r**_1_ to a point **r**_2_ can be described in terms of the Huygens−Fresnel principle in the frequency domain as a integral by integration over the plane Σ_1_ perpendicular to the *z* axis (beam axis): 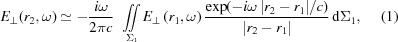
where ω is the angular frequency of the wave and *c* the speed of light. The wavefield at the observation plane can be written in a more general form as 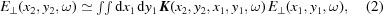
where the kernel ***K***(*x*_2_, *y*_2_, *x*_1_, *y*_1_, ω) is given as 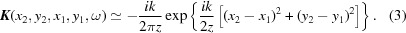



As shown in the following two sections, propagation through most X-ray optics elements can be presented in the form of the convolution integral (2). Using the Fourier optics modular approach, a complete beamline can be described as a set of propagators corresponding to individual optical components, which can be then numerically solved by means of a two-dimensional FFT algorithm.

The representation (3) also enables the analytical treatment of the quadratic phase term, allowing for free-space propagation simulations with a reduced sampling rate (Chubar *et al.*, 2008 ▸). This dramatically improves the technical feasibility and robustness of the Fourier optics method.

### 3.1. Thin optical elements   

Many of the optical components of an X-ray beamline can be described as thin optical elements, *i.e.* as linear filters that change the wavefield amplitude and/or phase in the plane normal to the propagation direction. For thin optical elements the kernel (3) can be represented as 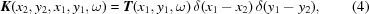
where ***T***(*x*_1_, *y*_1_, ω) is a complex transmission function, and δ() is the δ function. For example, the transmission function for a thin lens is given by 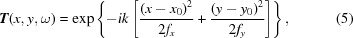
where (*x*_0_, *y*_0_) are coordinates of the lens center, and *f_x_* and *f_y_* are focal distances in the horizontal and vertical planes. The transmission for a two-dimensional parabolic compound refractive lens (CRL) (Kohn *et al.*, 2003 ▸) is given by 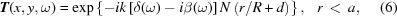
where the complex refractive index of the lens material is *n* = 1 − δ + *i*β, the CRL thickness depending on the distance from the lens center 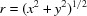 is *t* = *N* (*r*/*R* + *d*), *R* is the radius of the parabola tip, *N* is the total number of individual parabolic lenses, *d* is the minimum spacing between two parabolas and *a* is the lens aperture.

### 3.2. Extended optical elements   

The wave optics method that we use for simulation of grazing-incidence mirrors is based on the local stationary-phase approximation (Canestrari *et al.*, 2014 ▸). The kernel is presented as 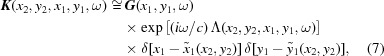
where ***G*** is a matrix function defining local transformations of amplitudes of electric field components, *i.e.* the propagation between input (*x*_1_, *y*_1_) and output (*x*_2_, *y*_2_) planes including the reflection from a mirror surface; 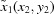 and 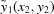 are scalar functions defining the transformation of coordinates for points in transverse planes before and after the optical element; and Λ(*x*_2_, *y*_2_, *x*_1_, *y*_1_, *ω*) is a scalar function defining the optical path between the points in the input and output planes. These functions can be found by using the asymptotic expansion of the Fresnel−Kirchhoff integral over the mirror surface, namely, the stationary phase approximation, which is Fermat’s principle for mirror reflection expressed in mathematical form. The stationary phase point can be found by defining a ray from the point (*x*_1_, *y*_1_) in the input plane in the direction provided by the local gradient of the input radiation field phase, finding its intersection with the mirror surface, generating the specular reflected ray, and finding its intersection with the output plane at the point (*x*_2_, *y*_2_) behind the mirror. As a final step, the electric field components are re-calculated on the required rectangular mesh, using a two-dimensional interpolation.

For gratings (Canestrari *et al.*, 2014 ▸), an extra phase shift ΔΦ(*x*_2_, *y*_2_, ω) associated with the reflection should be taken into account: 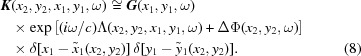
This function describes the phase shift introduced by grooves in the transverse direction, the shift being perpendicular to the grooves in the output plane: 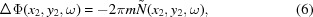
where *m* is the diffraction order, and 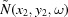 is the effective groove number for a given intersection point. For a variable line spacing (VLS) grating with a groove density that varies along the ‘longitudinal’ direction of the grating surface according to the *n*th order polynomial 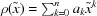, the effective number of grooves associated with the longitudinal position 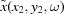 on the grating surface is 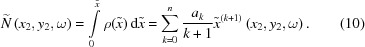



Propagation of the XFEL pulses through a perfect diffracting crystal can be described within the Fourier optics approach (Bushuev, 2008 ▸). For that, the crystal propagator known from dynamical diffraction theory is applied to wavefield Fourier amplitudes in reciprocal space. Afterwards the resulting wavefront in real space can be obtained with an inverse Fourier transformation. For implementation details see Sutter *et al.* (2014 ▸); the first practical applications of these *SRW* modules were described by Suvorov *et al.* (2015 ▸) and Chubar *et al.* (2016 ▸).

The available optical elements are listed in Table 1 ▸.

### 3.3. Imperfections in optics   

High-performing X-ray mirrors must be able to preserve the wavefront of the incident radiation inside the focused spot on the sample. Deviation of the mirror surface from an ideal one results in the appearance of scattered waves, which form speckles (irregularities of the radiation intensity caused by irregularities of the wavefront) in the beam spot downstream. In particular, the XFEL radiation divergence is only several microradians, and the source-to-mirror, *r*_0_, and the mirror-to-sample, *r*_1_, distances range up to several hundred metres. As a result, the mirror surface errors at the longest spatial wavelengths, comparable to the mirror length, have the greatest effect on the quality of the reflected beam. The spatial frequencies responsible for scattering radiation inside the spot (damaging frequencies) for a flat mirror, *e.g.* horizontal offset mirrors at the European XFEL, can be estimated as (Yashchuk *et al.*, 2015 ▸)
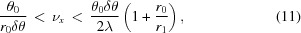
where θ_0_ is the incidence angle, δθ is the radiation divergence and λ is the X-ray wavelength. With the parameters of the SASE1 beamline at the European XFEL, the range of spatial frequencies that can produce speckles is ν*_x_* ≃ 0.033–0.3 cm^−1^. These frequencies correspond to the spatial lengths *d_x_* ≃ 3.3–30 cm. Lower spatial frequencies (spatial lengths longer than 30 cm) would lead to splitting of the beam. With the parameters of the SASE3 beamline, the range of spatial frequencies that will generate speckles is ν*_x_* ≃ 0.02–1.5 cm^−1^. These frequencies correspond to the spatial lengths *d_x_* ≃ 0.67–50 cm.

If the coherent X-ray beam is reflected at grazing angle θ from a very smooth mirror surface with a height profile *h*(*x*_m_), the optical path differences introduced by the mirror’s imperfections are much smaller than the X-ray wavelength λ: 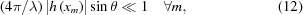
where *x*_m_ is the coordinate along the mirror surface and *m* is the point number. In this case one can use the complex transmission function 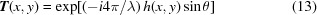
to analyze wavefront distortions induced by mirror surface residual height errors. Owing to the very small incidence angle the beam footprint is much larger along the propagation direction than in the sagittal direction. If the full width at half-maximum of the XFEL beam is about 0.5 mm, for a typical incidence angle 2 mrad, the beam footprint is about 25 × 0.5 mm. Since the impact of sagittal errors will be small, one can use here only one-dimensional metrology data to model the surface imperfections along the beam propagation direction.

The propagator of an imperfect CRL is simulated in a two-step approach. First, it uses the propagator for an ideal CRL as given in §[Sec sec3.2]3.2. Then, voids are simulated respecting a given void size distribution between a minimum and a maximum void diameter and are distributed in accordance with a given void density into a fictive cylinder of the same material, having the same aperture and thickness as the ideal CRL. In the next step, the paraboloids that are defining the CRL shape are removed, including the voids there. Finally, a plain void propagator is generated with the corresponding transmission. The passage of the wavefront through an imperfect CRL is thus calculated with two propagators, first for the ideal CRL, followed by the plane void propagator. To speed up the calculations the propagators for perfect and imperfect CRLs can be calculated only once, saved in a binary format supported by Python and later loaded from the hard drive. See also Roth *et al.* (2014 ▸), example 7 (https://github.com/ochubar/SRW), and the *WPG* tutorial (http://wpg.readthedocs.org/en/latest/tutorials.html).

### 3.4. Wavefront propagation setup   

**Figure 1:**
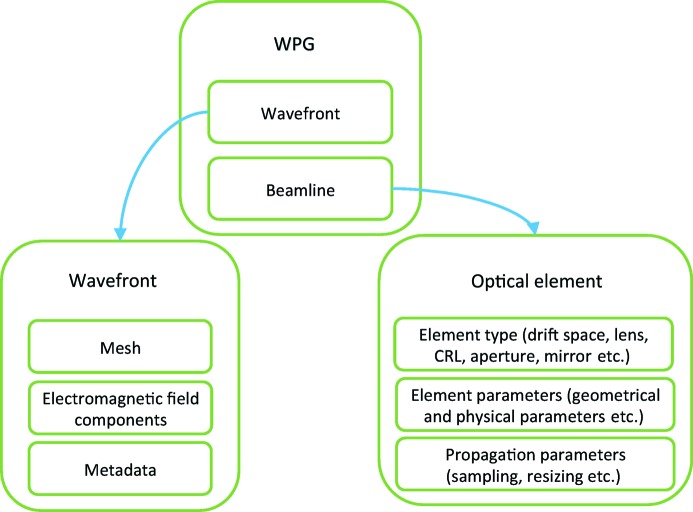
General structure of the *WPG* framework.

In the *WPG* framework the model of wavefront propagation is implemented using two classes, Wavefront() and Beamline() as shown in Fig. 1 ▸. To start the propagation one should define a wavefront, which can be a Gaussian steady state or time-dependent short pulse calculated using corresponding library functions, or an external XFEL pulse, *e.g.* from the X-ray FEL Photon Pulses Database (XPD; Manetti *et al.*, 2016 ▸), or any other external wavefield defined on a uniform Cartesian grid.

**Figure 2:**
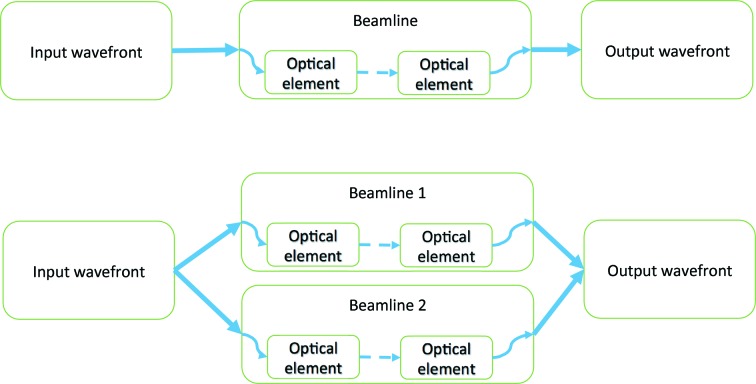
Workflow chart: a wavefront propagates through a beamline, which is set up as a container of propagators. Top: a simple ‘linear’ workflow for a regular beamline; bottom: beamline with two branches, *e.g*. XFEL split-and-delay lines.

The beamline is defined as a container of propagators with suitable parameters. The basic workflow is shown in Fig. 2 ▸.

#### 3.4.1. Wavefront data   

The structure of the HDF5 wavefront file, which is described in glossary file wpg/glossary.py, is used for mapping the Python wavefront structure to HDF5.

The *WPG* wavefront HDF5 file contains the obligatory and optional sections described below.

The main groups include the following:

(*a*) *data* – contains two three-dimensional arrays of electromagnetic fields with horizontal and vertical polarizations, arrEhor and arrEver

(*b*) *params* – contains a Cartesian grid of wavefront data, geometrical parameters *etc.*

The following are optional groups, especially recommended for use in start-to-end simulations:

(*a*) *history* – contains a link to a parent wavefront, if it exists, and a hierarchy structure with parent wavefront parameters and a link to its data

(*b*) *info* – some information about the origin of the current wavefront

(*c*) *misc* – information for visual checking of wavefront parameters (intensity distribution *etc*.)

(*d*) *version* – version of the wavefront glossary

#### 3.4.2. Beamline as a container of propagators   

**Figure 3:**
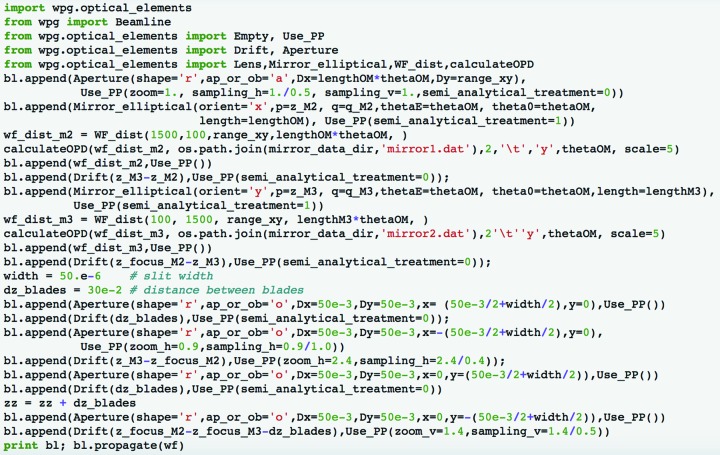
Example of a beamline definition: the SASE3 beamline at the European XFEL will include two horizontal offset mirrors (M1 and M2), a vertical focusing mirror M3, and horizontal and vertical clean-up slits.

An example of the beamline definition is shown in Fig. 3 ▸.

#### 3.4.3. Adjustment of propagation parameters   

Our framework provides special tools to facilitate propagation parameter adjustment and make the process transparent for the user. In particular, the method Use_PP() takes none or several propagation parameters defined by the user for a given optical element as arguments (see Fig. 3 ▸) and provides the core library propagation function with a full propagation parameter set, using for the rest the default values. For instance, bl.append(Drift(50.),Use_PP(semi_analytical_treatement=1)) introduces a 50 m drift in free space and sets up calculations using the semi-analytical processing described in §3. To tune the sampling, one can use the parameters zoom and sampling (Fig. 3 ▸). As in all numerical methods involving discrete Fourier transforms, the correct sampling of the signal in real and reciprocal space is critical for correct numerical calculations (see *e.g.* Potter, 1973 ▸; Goodman, 2004 ▸). To control the choice one can refer to the sampling in reciprocal *Q* space: the angular spectrum can be visualized using corresponding library utilities. See also the *WPG* documentation section with FEL beamline examples (http://wpg.readthedocs.org/en/latest/real_beamlines.html).

## 4. Examples   

### 4.1. FEL pulse visualization   

**Figure 4:**
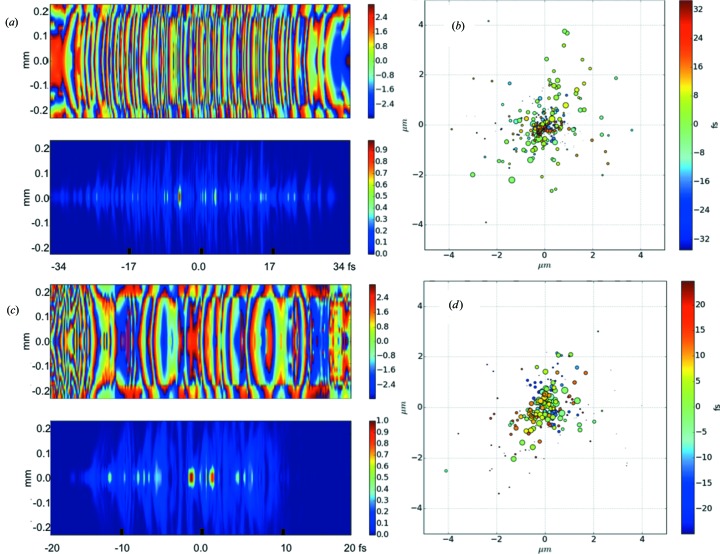
An example of three-dimensional wavefront visualization. For the SASE3 undulator with a defined maximum active segment length of 130 m, the simulated XFEL pulse data taken from the XPD (Manetti *et al.*, 2016 ▸) are used: photon energy 800 eV, electron bunch charge 250 pC and electron energy 14 GeV. The top row corresponds to an untapered undulator and pulse energy 8 mJ, and the bottom row to an undulator with optimized tapering (Schneidmiller & Yurkov, 2016 ▸) and pulse energy 17 mJ. (*a*), (*c*) Vertical cuts of the pulse phase and intensity; (*b*), (*d*) slice-to-slice shift of the pulse center of mass. The slice times are color coded and the intensities are represented by the size of the circles.

Fig. 4 ▸ shows the intensity and phase distribution of an XFEL pulse extracted from the XPD (Manetti *et al.*, 2016 ▸) and visualized by the *WPG* package at the exit of the SASE3 undulator with a maximum length of active segments of 130 m. The FEL data were simulated with the *FAST* code (Saldin *et al.*, 1999 ▸) for photon energy 800 eV, electron bunch charge 250 pC and electron energy 14 GeV. Note that the shift is much smaller than the spot size. One can easily observe that the pulse after the tapered undulator is shorter and more collimated.

### 4.2. Modeling the SCS SASE3 beamline   

**Figure 5:**
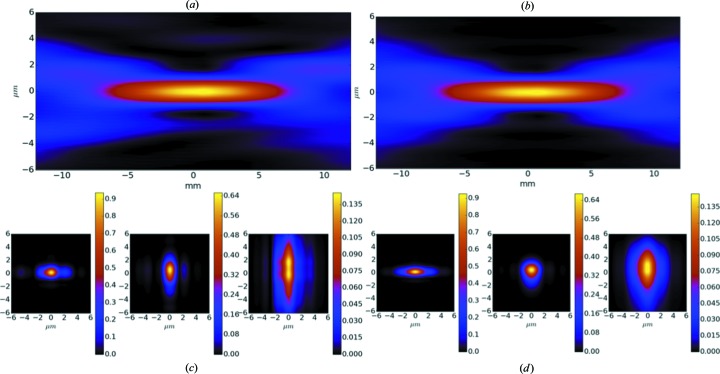
Intensity distribution around the sample position of the SCS instrument. Left panel (*a*), (*c*): the clean-up slits in the vertical and horizontal focus are fully open. Right panel (*b*), (*d*): both clean-up slits are closed to a gap of 50 µm. The cuts perpendicular to the optical axis in (*c*) and (*d*) correspond to −6, 0 and 6 mm positions around the focus.

Fig. 5 ▸ shows the intensity distribution around the sample position of the SCS beamline at the European XFEL. The optical elements considered in this example are offset and distribution mirrors of the beam transport, vertical mirrors of the soft X-ray monochromator, clean-up slits in the vertical and horizontal intermediate foci, and Kirkpatrick–Baez micro-focusing mirrors close to the sample position.

Cut-off effects of all optical elements were considered, assuming here an idealized Gaussian beam profile as a source with a divergence corresponding to XFEL SASE3 radiation from electron bunch charge 100 pC and electron energy 17.5 GeV. The simulations took into account a slightly deteriorated performance of the beamline mirrors with respect to their specifications. The left panel of Fig. 5 ▸ shows the beam intensity around the focus position with the clean-up slits fully open. The vertical cuts correspond to (left to right) −6, 0 and 6 mm positions around the focus. Asymmetric wings are visible in the horizontal plane, while the focus size is slightly enlarged in the vertical plane. By closing the clean-up slits (right panel), the focus becomes more homogeneous and smaller in the vertical plane. As demonstrated in this example, clean-up slits can in principle be used to reduce the effect of profile distortions from upstream mirrors without losing too much intensity. However, damage effects on these slits have to be carefully monitored and make this equipment usable only for moderate X-ray beam intensities.

### 4.3. Module for start-to-end simulation of XFEL experiments   

The *prop* module numerically propagates the FEL wavefield data from the undulator exit, through the beamline optics, to the sample position using the Python module *multiprocessing*. Each file from the working directory is processed by one free CPU core. On modern PCs or servers this makes it possible to significantly speed up the calculations. However the memory load by every process can become a bottleneck: the required memory for processing of a short 30 fs XFEL pulse can exceed 8 Gb for a beamline including submicrometre focusing optics, such as the Kirkpatrick–Baez mirror system of the SPB-SFX instrument at the European XFEL (Bean *et al.*, 2016 ▸). The module was successfully used for modeling of a single biomolecule imaging experiment at the SPB instrument with the multiphysics simulation framework *simS2E* (Yoon *et al.*, 2016 ▸).

## 5. Software availability and documentation   

The *WPG* framework runs reliably under Linux, Microsoft Windows 7 and Apple Mac OS X. Using IPython as a web front-end enables the users to run the code on a remote server as well as on their local personal computers. One can use popular Python libraries [such as *SciPy* (https://www.scipy.org/), *NumPy* (http://www.numpy.org/) and *Matplotlib* (http://matplotlib.org/)] for pre- and post-processing as well as for visualizing the simulation results.

The wavefronts are saved in HDF5 format for eventual further processing and start-to-end simulations of experiments. The HDF5 format allows for keeping the calculation history within a single file, thus facilitating communication between various scientific groups and cross-checking with other simulation results. The *WPG* source code (https://github.com/samoylv/WPG) together with guidelines for installation and application examples (https://wpg.readthedocs.org/en/latest/) are open source and available on the web. The installation includes building the C++ written *SRW* library from its sources.

The installation procedure for Linux, OS X and Windows 7 is described at https://wpg.readthedocs.org/en/latest/wpg.html#getting-started and includes installation of Python, *Numpy*, *Scipy*, *Matplotlib*, *h5py* (http://www.h5py.org/) and the *IPython Notebook*.

## 6. Future steps   

In the near future, we plan to enhance the start-to-end simulation possibilities by employing the MPI technology through the *mpi4py* module and running the propagation of multiple pulses on a cluster. One should, though, keep in mind that transferring the input and resulting pulse data through the network can also become a bottleneck. Thus the efficiency strongly depends on the cluster configuration and the ratio of calculation time for one pulse and network data transfer rate. In addition, we plan for the *WPG* to support full inter-operation with a new framework, based on client–server architecture and using JavaScript and Python for a rich graphical user interface. This is currently under development in a project (Bruhwiler *et al.*, 2014 ▸) sponsored by the US Department of Energy.

## 7. Conclusions   

Knowledge of temporal, spatial, spectral and coherence properties of the radiation from X-ray FELs is of key importance for planning user experiments. We have developed the *WPG* software package as a new framework for wave optics simulations and have used it successfully for X-ray FEL optics beamline design and experimental data analysis. The software is also used as a part of the multiphysics simulation framework *simS2E* for source-to-experiment simulations of a single-particle imaging experiment, employing pulse data from the XPD as an input. For the upcoming commissioning of the European XFEL, the *WPG* will be available as a versatile tool capable of simulating all relevant optics at the facility.

## Figures and Tables

**Table 1:** Optical elements (propagators) available in the *WPG* The parameters are shown in ***bold italic*** and the names of the propagators in *italic*.

Optics	Propagator	Parameters	Comments
Free space	*Drift*	***Propagation distance***	In most cases the semi-analytical propagation is recommended, see §3.1.
Slits, apertures	*Aperture*	***Slit or obstacle***, ***shape*** (rectangular or circular), ***slit width***, ***height***, ***slit center coordinates***	For a circular aperture only slit width is used as aperture diameter.
Grazing-incidence plane elliptical mirror	*Mirror_elliptical*	***Orientation***, ***p*** and ***q***, distances to source and focus, ***incidence angle*** in the mirror center, ***misalignment angle***, ***mirror length***	See §3.3 for details.
Thin lens	*Lens*	***Focal lengths*** in horizontal and vertical plane, and ***lens center coordinates***	Can be used to model sagittal bending in a plane elliptical mirror or to approximate focusing with a spherical mirror.
Mirror surface error†	*WF_dist*	***Mesh*** and ***dimensions***	Phase screen approach is used to introduce wavefront distortions caused by residual surface height errors (Samoylova *et al.*, 2009 ▸).
Grazing-incidence VLS grating	*VLS_grating*	***Substrate*** (a mirror propagator), ***diffraction order number m***, ***grove density g*_0_** in the grating center, ***g_k_***, ***k* = 1,…, 4**, ***grove density polynomial coefficients***: *g* = *g*_0_ + *g*_1_ *x* + *g*_2_ *x* ^2^ + *g*_3_ *x* ^3^ + *g*_4_ *x* ^4^	See §3.3 for details.
Crystal monochromator	*Xtal*	***Interplanar spacing*** of the selected Bragg reflection *H*, ***Fourier components of electric susceptibility***,‡ crystal ***thickness***, ***asymmetry angle***, ***deviation of the incidence angle*** from the rocking curve center	
CRLs	*CRLs*	Obligatory parameters: ***which focal plane*** (1 for horizontal, 2 for vertical, 3 for both), ***δ***, ***attenuation length***, ***shape*** (parabolic or spherical), horizontal and vertical ***aperture diameters***, ***tip radius***, ***number*** of CRLs (holes), ***lens thickness at apex***; optional parameters: a flat array/list of ***void center coordinates and radii***: [*x*_1_, *y*_1_, *r*_1_, *x*_2_, *y*_2_, *r*_2_,…], ***initial photon energy***, ***final photon energy***	See also §3.3 on modeling voids in CRLs. Note: the energy range parameters are used only if the attenuation length and *δ* are arrays. Then the dispersion effect of CRLs is taken into account.

† The propagator should be used together with the calculateOPD() function, see Fig. 3 ▸.

‡ For *0*, *H* and −*H* reciprocal lattice nodes for reflection *H*.
